# Polyelectrolyte Membrane Nanocoatings Aimed at Personal Protective and Medical Equipment Surfaces to Reduce Coronavirus Spreading

**DOI:** 10.3390/membranes12100946

**Published:** 2022-09-28

**Authors:** Anna Grzeczkowicz, Agata Lipko, Angelika Kwiatkowska, Marcin Strawski, Paweł Bącal, Agnieszka Więckowska, Ludomira H. Granicka

**Affiliations:** 1 Nalecz Institute of Biocybernetics and Biomedical Engineering, Polish Academy of Sciences, Trojdena 4 st., 02-109 Warsaw, Poland; 2 Laboratory of Electrochemistry, Faculty of Chemistry, University of Warsaw, Pasteura 1 st., 02-093 Warsaw, Poland; 3 Institute of Paleobiology, Polish Academy of Sciences, Twarda 51/55 st., 00-818 Warsaw, Poland; 4 Department of Inorganic and Analytical Chemistry, Faculty of Chemistry, University of Warsaw, Pasteura 1 st., 02-093 Warsaw, Poland

**Keywords:** COVID-19, polyelectrolyte membranes, nanoparticles, medical equipment surfaces, personal protective equipment

## Abstract

The study of the surface of membrane coatings constructed with adsorbed coronavirus (COV) was described to test their suitability for the antiviral activity for application in personal protective and medical equipment. The nanocoating based on polyethyleneimine (PEI) or polystyrene sulfonate (PSS) with metallic nanoparticles incorporated was investigated using the AFM technique. Moreover, the functioning of human lung cells in a configuration with the prepared material with the adsorbed coronavirus was studied using microscopic techniques and flow cytometry. The mean values of the percentage share of viable cells compared with the control differed by a maximum of 22%. The results showed that PEI and PSS membrane layer coatings, modified with chosen metallic nanoparticles (AuNPs, AgNPs, CuNPs, FeNPs) that absorb COV, could support lung cells’ function, despite the different distribution patterns of COV on designed surfaces as well as immobilized lung cells. Therefore, the developed membrane nanocoatings can be recommended as material for biomedical applications, e.g., medical equipment surfaces to reduce coronavirus spreading, as they adsorb COV and simultaneously maintain the functioning of the eukaryotic cells.

## 1. Introduction (Semi-Review)

In 2020, the world faced a global coronavirus disease 2019 (COVID-19) pandemic. The sudden increase in cases has paralyzed health care in many developed countries. In response to the crisis, governments closed borders and limited the flow of people, hoping to stop the virus from spreading. Unfortunately, these restrictive means affected industries and economies [1,2], causing the inhibition of the supply chains and impacting many societies’ lives [3]. The most challenging has been to reduce the contact between people. Thus, isolation, social distance, and protective clothing were applied as prevention [4]. Nonetheless, these non-pharmaceutical interventions were unreliable in some areas of social life. After over two years of everyday life in the pandemic’s shadow, procedures have been elaborated to minimize virus transmission risk [5]. However, the resources at our disposal are still insufficient to say that society can provide adequate protection to people working in conditions of increased risk. Moreover, based on the historical data, it can be concluded that the current one is not the last pandemic that humankind must face [3,6]. Therefore, working on materials supporting the reduction of viral transmission is an ongoing issue.

The virucidal properties of designed surfaces correlate to the virus attachment mechanism and host cell entering. There are materials of both natural and synthetic origins, commonly considered antiviral; among them, metals, graphene, peptides, and polymers can be enumerated. Although not many studies have been performed on the antiviral activity of surfaces against SARS-CoV-2, it is assumed that the materials that exhibit viricidal properties against other coronaviruses should also be effective in that case/in the battle against COVID-19. A good example might be metal ions such as copper and silver, especially Cu ions, which can interact with viral components by inactivating their metalloproteins. It was reported that cooper and cooper alloy surfaces inactivate coronavirus, leading to irreversible and nonspecific RNA fragmentation [7]. Polymers are another interesting group of materials showing antiviral properties, also against SARS-CoV-2. Among them synthetic (poly-L-lysine—PLL [8], diethylaminoethyl-dextran—DEAE-dextran [9,10], poly(dl-lactide-co-glycolide)—PLGA [11], polyethyleneimine—PEI [12], poly(amidoamine)—PAMAM dendrimers [12]) and natural (carrageenans [13], cyclodextrin [14,15,16], chitosan [17]) polymers can be listed [10,18]. Another antiviral material which gained great popularity recently is graphene. Graphene derivatives, especially graphene oxide (GO) and reduced graphene oxide (rGO), have exhibited a wide-spectrum inhibitory effect against bacteria, fungi, and viruses [19].

Although the materials mentioned above interact with virus particles differently, the Rakowska group distinguishes six main mechanisms of action, i.e., (1) inactivating surfaces (e.g., by binding to the capsid proteins or membrane; material: biopolymers); (2) controlled release of virucides; material: AMPs); (3) producing ROS; photosensitizing materials; (4) RNA/DNA degradation (e.g., by ionic surfaces; material: metals, polyethyleneimines); (5) membrane disruption by dehydration (e.g., by adsorbing surfaces); (6) membrane disruption by puncture (e.g., by sharp nanostructured surfaces; material: graphene) [10].

There are many reports of the successful application of layer-by-layer nanocoatings on the design of antimicrobial surfaces. Polyelectrolyte membranes, a type of material in high demand in health and environmental sectors, can play an essential role in this purpose [20,21,22,23].

The exciting approach that might be the next step of the layer surfaces evolution is to incorporate the nanoparticles of antiviral activity with nano-thin layers of polyelectrolytes. The nanocoatings of such a type might potentially serve as an outer layer of materials dedicated for usage in public areas or might be applied for protective clothing production.

Herein, we present the designed nanocoatings of antiviral activity for application in personal protective and medical equipment.

The different synthetic polymers were assessed for that purpose concerning biocidal activities. For example, the presence of sulfonate functional groups in evaluated block polymers was claimed to exert biocidal activity, among others, against Influenza A [24]. Moreover, biocidal activity was exhibited by PEI [25,26]. The neutral PEI was not reported to exert antiviral activity; we decided to consider the PEI and polystyrene sulfonate (PSS) nanocoatings mentioned above—the materials for building the membrane with respectively a mildly positive and negative potential—via a usability examination. The chosen metallic nanoparticles had membrane coatings incorporated.

Moreover, the paper presents the results of interaction with human lung cells of the adsorbing COV material built of polyelectrolytes with selected NPs incorporated, which has not been reported before.

## 2. Materials and Methods

### 2.1. Materials

*Reagents*: poly(ethyleneimine), branched, Mn ~ 60,000, Mw 750,000, analytical standard, 50% (*w*/*v*) in H_2_O (Sigma-Aldrich, Munchen, Germany); poly(sodium 4-styrene sulfonate), Mw ~ 70,000, (Sigma-Aldrich, Munchen, Germany); propidium iodide (Sigma-Aldrich), trypsin EDTA solution C (0.5%), EDTA 0.2% (10×) (Biological Industries, Kibbutz Beit HaEmek, Israel), phosphate-buffered saline (PBS) (Biomed Lublin, Lublin, Poland), MilliQ water; silver nanoparticles, 100 ppm, of 10 nm size, stabilized with sodium citrate, in 0.01% Tween 20 (University Technology Transfer Centre of the University of Warsaw (UTTC UW): Bell Synthesis, Poland, EU); golden nanoparticles, 100 ppm, of 10 nm size, stabilized with sodium citrate, in 0.01% Tween 20 (UTTC UW: Bell Synthesis, Warsaw, Poland); copper nanopowder 25 nm particle size (Merck/Sigma-Aldrich); iron (II) chloride tetrahydrate (Sigma Aldrich, St. Louis, MO, USA); iron (III) chloride hexahydrate (Sigma Aldrich, St. Louis, MO, USA).

*Media*: Ham’s F12 Medium/Dulbecco’s Modified Eagle’s Medium (F12/DMEM) (Gibco, Thermo Fisher Scientific, Waltham, MA, USA); Fetal Bovine Serum (FBS) (Sigma).

*Culture medium*: F12-K medium (Kaighn’s Modification of Ham’s F-12 Medium) supplemented with 10% FBS (Gibco, Thermo Fisher Scientific, Waltham, MA, USA). *Cells*: Human adenocarcinoma A549 cell line from human lung.

### 2.2. Methods

#### 2.2.1. FeNPs Synthesis

FeNPs synthesis: Iron nanoparticles, Fe_3_O_4_NPs, (FeNPs), were synthesized using a co-precipitation method. First, 2.703 g of iron(III) chloride hexahydrate (FeCl_3_∙6 H_2_O) and 0.994 g of iron(II) chloride tetrahydrate (FeCl_2_∙4 H_2_O) were dissolved in 50 mL of deionized water (Fe^3+^:Fe^2+^ in 2:1 molar ratio). Then, 10 mL of a precipitant—a 25% wt solution of NH_4_OH—was added to the iron chlorides. Next, the mixture was stirred for 30 min at room temperature. After that time, the dark brown-black iron nanoparticles were formed. The FeNPs were rinsed with 200 mL of deionized water three times, then centrifuged at 10,000 rpm for 15 min. Finally, the supernatant was removed.

#### 2.2.2. Preparation of Polyelectrolyte Membranes Deposited on Polystyrene Support for SEM/TEM Studies

The bottoms cut from the 96-well culture plate were soaked overnight in 75% ethanol, then rinsed in sterile deionized water. After drying the bottoms, they were placed in the following solutions for half an hour: (1) PSS at concentration 1 mg/mL in PBS, (2) PEI at concentration 1 mg/mL in PBS. The next step was to dry the bottoms (overnight). The next day, the bottoms were placed in the wells of a 6-well plate.

The suspension containing the dead Vero 6 cells infected with the heat-inactivated SARS-CoV-2 virus (Heat-inactivated SARS-CoV-2 (ATCC^®^ VR-1986HK™) (COV) was centrifuged for 20 min (Eppendorf mini spin; max speed—13.4 × 1000 rpm, 12,100× *g* rcf). Next, for each previously prepared bottom covered with polyelectrolyte membranes, 15 µL of the supernatant was poured. The suspension was spread over the entire available surface if possible. The samples were left under the laminar for 30 min. Then the samples were quenched with glutaraldehyde and left for the next 30 min. Finally, the samples were dehydrated using the alcohol series (50%, 70%, 90%, and 100% after 15 min) and left to dry.

#### 2.2.3. Preparation of the Polyelectrolyte Membranes

The following membranes based on polyethyleneimine were prepared:

(1) Polyethylenimine (PEI) incorporating AuNPs (PEI-Au),
(2) Polyethylenimine incorporating AgNPs (PEI-Ag),
(3) Polyethylenimine incorporating CuNPs (PEI-Cu),
(4) Polyethylenimine incorporating FeNPs (PEI-Fe).

Membranes were obtained by adding 10 ppm of nanoparticles water solutions (AuNPs, AgNPs, CuNPs, and FeNPs, respectively) to a 1 mg/mL PEI solution in PBS at a 1:1 ratio, followed by the mixture stirring for 4 h at room temperature.

The following membranes based on polystyrene sulfonate were prepared:

(1) Polystyrene sulfonate (PSS) incorporating AuNPs (PSS-Au)
(2) Polystyrene sulfonate incorporating AgNPs (PSS-Ag)
(3) Polystyrene sulfonate incorporating CuNPs (PSS-Cu)
(4) Polystyrene sulfonate incorporating AgNPs (PSS-Fe)

10 ppm of nanoparticles’ water solutions (AuNPs, AgNPs, CuNPs, and FeNPs) were added to a 1 mg/mL PSS solution in PBS at a 1:1 ratio and subsequently stirred for 4 h at room temperature.

All the membranes were deposited on glass coverslips.

#### 2.2.4. A549 Cell Line Culture on Glass Coverslips Covered by Polyelectrolyte Membranes with Adsorbed COV

The human adenocarcinoma A549 cell line was maintained in an F12-K medium (Kaighn’s Modification of Ham’s F-12 Medium) supplemented with 10% FBS at 37 °C in a humidified 5% CO_2_/95% atmosphere. After trypsinization, cells (1 × 10^3^/cm^2^) were positioned on the polyelectrolyte membranes listed above with adsorbed COV as described above, deposited on glass coverslips placed in separate wells of the 24-well cell culture plate with the non-treated surface, and cultured in 0.5 mL of F12-K medium for 10 days (5% CO_2_, 37 °C). Cells were cultured in the same conditions on unmodified glass coverslips as a control. Moreover, the cells were cultured on the membranes listed above with adsorbed Protein A as a second control, mimicking an adsorbed bacterial material. After 3, 6, and 10 days of culture, the cell viability was examined with propidium iodide using flow cytometry. Furthermore, the morphology of immobilized cells was examined via scan electron microscopy (SEM).

#### 2.2.5. Fluorescence Staining

For fluorescence staining, cells immobilized within membranes deposited on glass coverslips after 10 days of culture were fixed in 4% paraformaldehyde (PFA) in PBS at room temperature (20 °C) for 15 min. Then, cell membranes were permeabilized using TRITON X100 detergent to allow dyes to penetrate individual cells. The next step was the addition of fluorochrome-conjugated phalloidin, staining F-actin. Phalloidin is a toxin isolated from the fungus Phylum Amanita (Amanita phalloides), which binds directly to filamentous actin (F-actin) found in large quantities in fibroblasts. Then DAPI, fluorochrome specifically staining DNA, was added to the cells to visualize single cells. Under UV light, cell nuclei stained with DAPI shows blue fluorescence. After three washes in PBS, cells were photographed using an Olympus IX70 fluorescence microscope. Blue DAPI fluorescence (λ = 460 ÷ 500 nm) and red phalloidin fluorescence (λ = 570 nm) were examined.

#### 2.2.6. Flow Cytometric Analysis

The A549 cells were analyzed using a Canto II flow cytometer (Becton Dickinson Immunocytochemistry Systems, Franklin Lake, NY, USA). The results were processed by the FACS Diva software system (Becton Dickinson, Franklin Lake, NY, USA). Evaluated objects were separated from other events based on the light scattering characteristics.

#### 2.2.7. MTT Assay

The (3-4,5-dimethylthiazol-2-yl)-2,5-diphenyltetrazolium bromide (MTT) assay was applied to assess cellular mitochondrial activity. For that purpose, after 3, 6, and 10 days of culture of the cells seeded on the membrane films, the MTT solution at the concentration of 5 g/L was added to the culture in a 1:10 dilution of the medium. Next, the cells were incubated for 2 h at 37 °C with 5% CO_2_. Afterwards, the solution was discarded, and DMSO was added to each well. After 15 min of shaking, absorbance was measured at 550 nm using a spectrophotometer (HP 8452 diode-array spectrophotometer).

#### 2.2.8. Scanning Electron Microscopy Analysis

SEM characterization was carried out with a Crossbeam 540X (Carl Zeiss Microscopy GmbH, Jena, Germany) scanning electron microscope with an X-FEG cathode. This microscope was applied to visualize the COV deposited on the membranes (the sample preparation is described above). Moreover, the cells immobilized within membranes were visualized using a scanning electron microscope (SEM); (TM 1000, Hitachi, Tokyo, Japan). After 3 and 10 days of culture, cells were fixed with 2.5% glutaraldehyde. Then, the fixed samples were rinsed several times with Milli Q water and placed for 15 min in 75.0% ethanol. The procedure was repeated. The next stage was a 15-min incubation of samples in 99.8% ethanol. Then, the samples were air-dried and placed on microscope tables.

#### 2.2.9. Atomic Forces Microscopy Evaluation

We employed atomic forces microscopy (AFM) to examine the prepared samples’ surface morphology. AFM examinations of the samples were performed using MultiMode 8 (Bruker, Billerica, MA, USA). Silicon cantilevers with a spring constant of ca. 5 Nm^−1^ (TapDLC-150, BudgetSensors, Sofia, Bulgaria) were applied for imaging in PeakForce TappingTM microscopy mode. The sample preparation procedure includes the application of 150 µL of solution on the clean mica plate, V1 grade (NanoAndMore GmbH, Wetzlar, Germany). After 10 min, the plates were rinsed with deionized water and dried under a gentle stream of argon. The data were acquired in Nanoscope 8.15 software and then analyzed with NanoScope Analysis 1.4 software (Wetzlar, Germany).

#### 2.2.10. Fourier Transform Infrared (FT-IR) Spectroscopy

The infrared spectra were recorded using the Nicolet iS50 FT-IR spectrometer (Thermo Scientific) with a DTGS detector. An iTR-attenuated total reflection accessory with diamond crystal was used for all experiments. All experiments were performed with a resolution of 4 cm^−1^, and 32 scans were taken for each sample. A 10 µL drop of solution was deposited in the crystal and left to dry under a gentle air stream.

#### 2.2.11. Statistical Analysis

All data are expressed as mean ± standard deviation (SD), wherein the mean values, standard deviations, and the significance of differences were calculated in the Statistica 7.1 software (TIBCO Software, Palo Alto, CA, USA). Values where *p* < 0.05 were assumed to be significant.

## 3. Results and Discussion

### 3.1. Studies of Virus Adhesion to the Developed Membranes

#### Scanning Electron Microscopy Analysis

**Figure 1:**
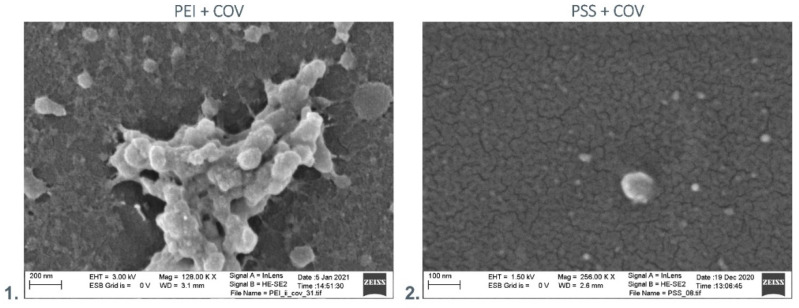
SEM image of COV deposited on the surface of the **1.** PEI membrane, **2.** PSS membrane.

The Scanning Electron Microscopy visualization was performed to examine the adsorption of the virus to the surface of the chosen membranes. As a result of COV adsorption to the PEI membrane surface, its morphology presents the shape of bunch-shaped structures. On the other hand, the COV adsorption to the PSS membrane surface implies flat morphology, with COV evenly dispersed all over the surface. In Figure 1, the COV deposited on the surface of the selected membranes is presented.

### 3.2. Analysis of the Functioning of Cells Immobilized within Membranes in the Virus Presence

We analyzed the functioning of cells immobilized within membranes maintained in the presence of the virus. The developed coatings with the adsorbed COV or Protein A were tested for cytotoxicity on the A549 human cell line in vitro during 10 days of culture. The immobilized cells were deposited on glass slides, wherein the cells cultured for 10 days in the presence of slides without membranes served as a negative control. Cell function and morphology were evaluated quantitatively by flow cytometry and qualitatively by SEM and fluorescent microscopy.

#### 3.2.1. Flow Cytometry

Flow cytometry results showed that after 10 days of A549 cells culture, the percentage of the viable cells cultured in the presence of PEI membrane with AuNPs incorporated alone or with adsorbed COV or Protein A (PROT A) was higher compared with control (*p* = 0.017; *p* = 0.005; *p* = 0.046 respectively). Similar results were found in the presence of membranes with incorporated CuNPs, without or with adsorbed COV or Protein A (*p* = 0.004; *p* = 0.034; *p* = 0.028 respectively), and AgNPs alone or with adsorbed COV. However, the difference in mean value did not exceed 22%. There was no significant difference in viable cell percentage between control and cells cultured in the presence of membranes with incorporated FeNPs, without or with adsorbed COV or Protein A (*p* = 0.203; *p* = 0.241; *p* = 0.782, respectively).

**Figure 2:**
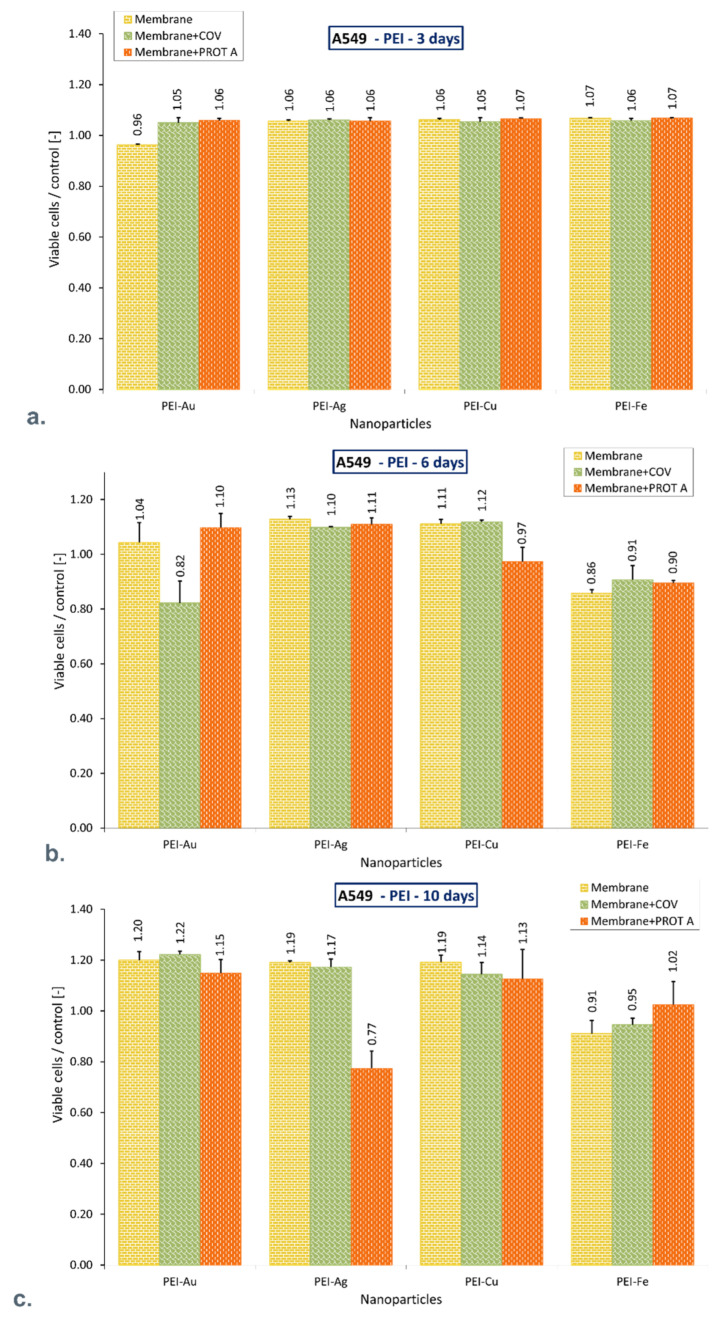
The ratio of the percentage of viable A549 immobilized on membranes to the control after (**a**) 3-day culture, (**b**). 6-day culture, (**c**). 10-day culture. The values are presented as mean ± SD. Key to the symbols: polyethyleneimine membrane with AgNPs incorporated (PEI-Ag), polyethyleneimine membrane with AuNPs incorporated (PEI-Au), polyethyleneimine membrane with CuNPs incorporated (PEI-Cu), polyethyleneimine membrane with FeNPs incorporated (PEI-Fe). Membrane—the membrane without adsorbed COV or PROT A; Membrane + COV—membrane with adsorbed COV; Membrane + PROT A—membrane with adsorbed Protein A.

**Figure 3:**
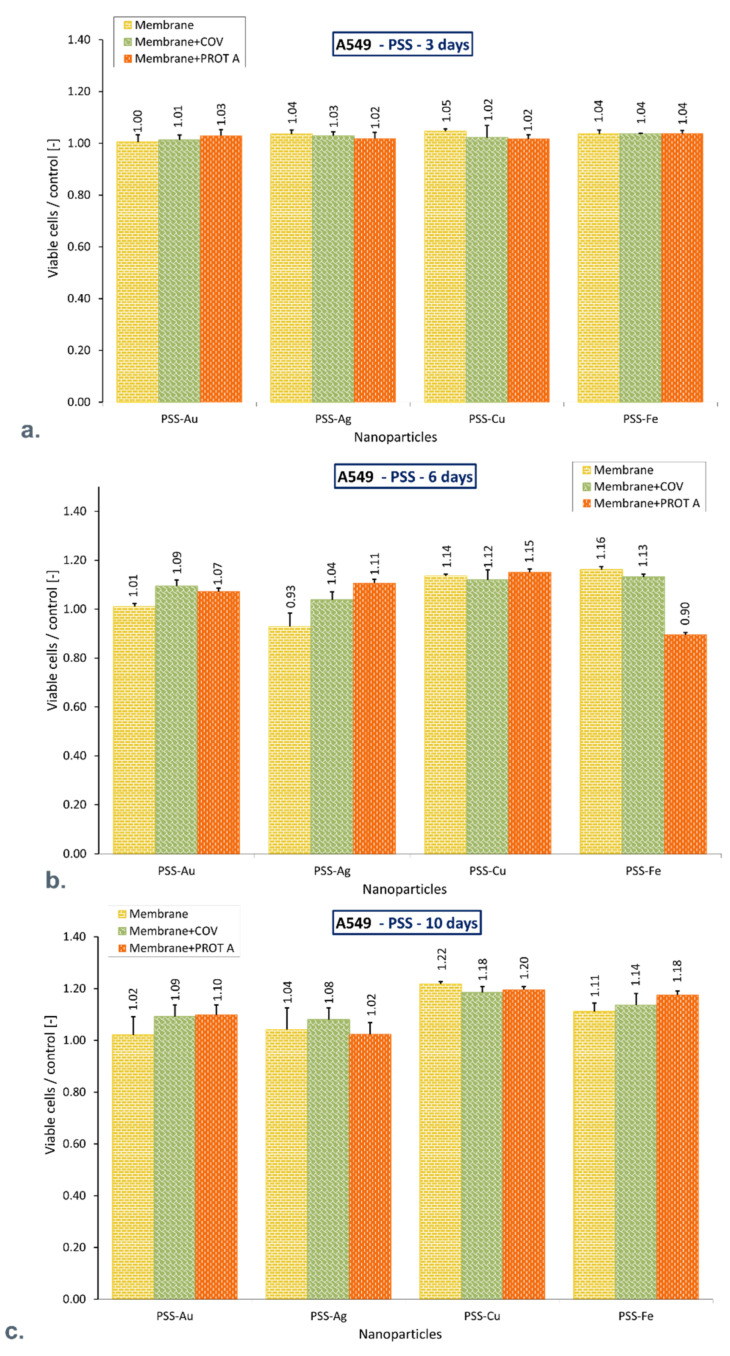
The ratio of the percentage of viable A549 immobilized on membranes to the control after (**a**). 3-day culture, (**b**). 6-day culture, (**c**). 10-day culture. The values are presented as mean ± SD. Key to the symbols: polystyrene sulfonate membrane with AuNPs incorporated (PSS-Au), polystyrene sulfonate membrane with AgNPs incorporated (PSS-Ag), polystyrene sulfonate membrane with CuNPs incorporated (PSS-Cu), polystyrene sulfonate membrane with FeNPs incorporated (PSS-Fe). Membrane—the membrane without adsorbed COV or PROT A; Membrane + COV—membrane with adsorbed COV; Membrane +PROT A—membrane with adsorbed Protein A.

However, the PEI-Ag + PROT A percentage share was lower than the rest of the membranes and did not exceed 23% compared to the control. Moreover, after 10 days of culture, in the presence of a PSS membrane with the different metallic nanoparticles incorporated without or with adsorbed COV or Protein A, the mean viable cell percentage value did not differ more than 22% compared to the control. The obtained results are presented in Figure 2 and Figure 3.

#### 3.2.2. Assessment of the Cell Mitochondrial Metabolic Activity Using MTT Assay

MTT analysis showed that COV and PROT A protein’s presence on the PEI-Au membrane adversely affects lung cells’ function after 3 days of culture. After culturing on membrane PEI-Au + COV and PEI-PROT A, a 30% and 70% decline in mitochondrial cell activity was observed, respectively, compared with control. However, after 10 days of culture, the membranes with adsorbed COV and PROT A promote the proliferation of cells like the PEI-Au membrane itself.

Nonetheless, PEI-Ag + COV caused a significant decline in mitochondrial activity; PEI-Ag + PROT A material did not adversely affect A549 cells after 3 days. On the other hand, after 10 days of culture, the mean value of mitochondrial activity ratio to the control was 28 ± 0.07%.

The PEI-Cu + COV and PEI-Cu + PROT A material causes reduced mitochondrial activity, although the PEI-Cu material maintains cells’ function after 10 days of culture. A 35% increase in mitochondrial activity of cells cultured on PEI-Cu was observed compared with control cells.

PEI-Fe and PEI-Fe + PROT A materials support cell function, increasing mitochondrial activity compared with control after 10 days of culture.

**Figure 4:**
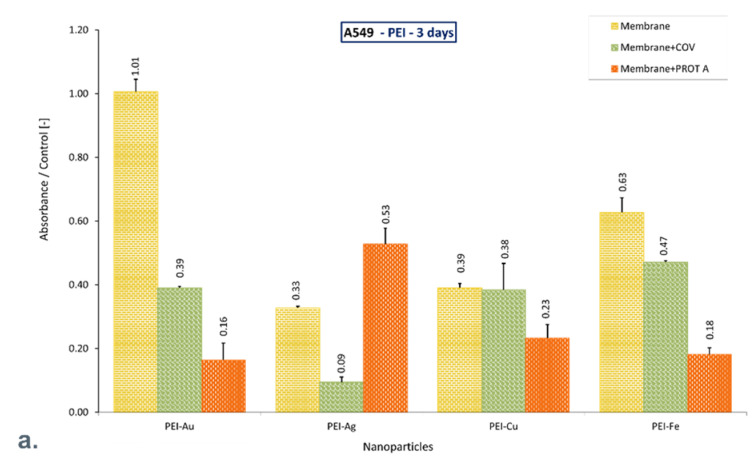

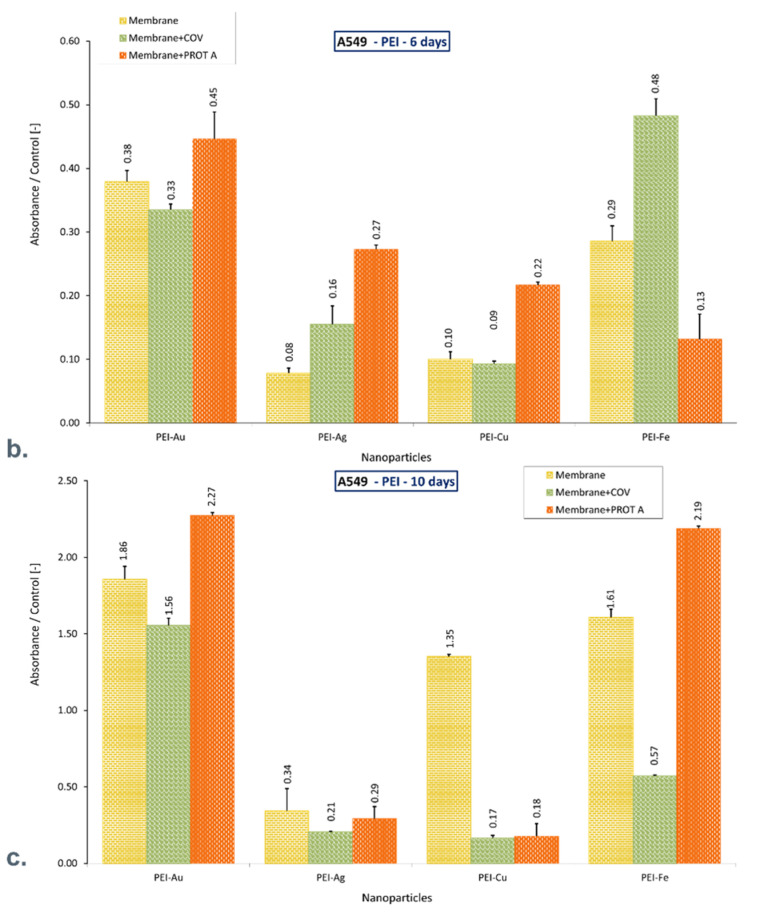
The ratio of the mitochondrial activity of A549 immobilized on membranes represented by formazan production expressed by absorbance to the control after (**a**). 3-day culture, (**b**). 6-day culture, (**c**). 10-day culture. The values are presented as mean ± SD. Key to the symbols: PEI-Ag: polyethyleneimine membrane with AgNPs incorporated; PEI-Au: polyethyleneimine membrane with AuNPs incorporated; PEI-Cu: polyethyleneimine membrane with CuNPs incorporated, PEI-Fe: polyethyleneimine membrane with FeNPs incorporated. Membrane—the membrane without adsorbed COV or PROT A; Membrane + COV—membrane with adsorbed COV; Membrane + PROT A—membrane with adsorbed Protein A.

It should be noted that after 10 days of culture, only the materials PEI with Au or Cu or Fe incorporated (PEI-Au, PEI-Cu, PEI-Fe) maintain cell function, exhibiting increased mitochondrial activity compared with control. Adsorption of COV in PEI-Au does not reduce the cells’ activity, whereas adsorption of PROT A on membrane PEI-Au or PEI-Fe increases mitochondrial activity twice. Moreover, adsorption of COV on these membranes causes over 50% increase or over 50% decrease, respectively, compared with control (Figure 4).

Despite the adsorbed COV and Protein A, the PSS-Au and PSS-Ag membranes allow the cells to retain function after 10 days of culture, increasing the mitochondrial activity compared to the control. Likewise, the PSS-Fe membrane preserves the function of cells that exhibit mitochondrial activity similar to that of the control. On the contrary, the PSS-Cu membrane alone and with COV or PROT A adsorbed causes a decrease in mitochondrial activity by approximately 70%, compared with the negative control after 10 days of culture. However, a significant increase was observed for these membranes on the third day of culture.

**Figure 5:**
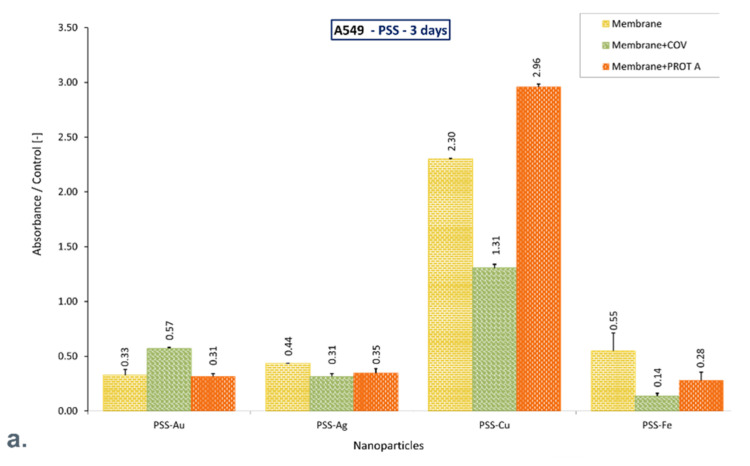

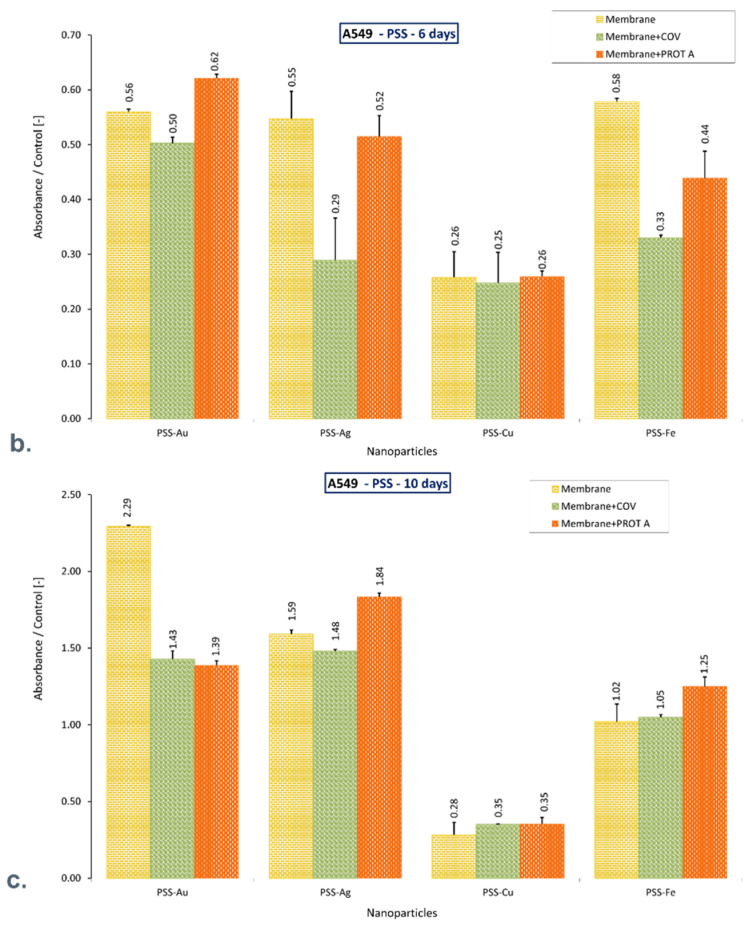
The ratio of the mitochondrial activity of A549 immobilized on membranes represented by formazan production expressed by absorbance to the control after (**a**). 3-day culture, (**b**). 6-day culture, (**c**). 10-day culture. The values are presented as mean ± SD. Key to the symbols: polystyrene sulfonate membrane with AuNPs incorporated (PSS-Au), polystyrene sulfonate membrane with AgNPs incorporated (PSS-Ag), polystyrene sulfonate membrane with CuNPs incorporated (PSS-Cu), polystyrene sulfonate membrane with FeNPs incorporated (PSS-Fe). Membrane—the membrane without adsorbed COV or PROT A; Membrane + COV—membrane with adsorbed COV; Membrane + PROT A—membrane with adsorbed Protein A.

It can be mentioned that after 10 days of culture, only materials based on PSS with Au or Ag, or Fe incorporated maintains cell function, exhibiting increased mitochondrial activity or comparable with control. Adsorption of COV or PROT A in these membranes does not decline the cells’ activity. On the contrary, adsorption of PROT A on membrane PSS-Ag increases mitochondrial activity almost twice (Figure 5).

Although applying AgNPs, AuNPs, CuNPs, and FeNPs at a concentration of 10 ppm incorporated in membranes did not negatively affect A549 cells in terms of their viability; one can observe the fluctuation of mitochondrial activity during the culture on the material surface, with different NPs incorporated dependently on the sort of NP and configuration with PSS or PEI material and time of culture. For example, PSS-Cu alone and with adsorbed COV or Protein A exhibits fluctuation compared with the control from higher mitochondrial activity on the third day to meanly 30% on the tenth day of culture. On the other hand, PSS-Ag alone and with adsorbed COV or Protein A, and PSS-Fe alone and with adsorbed COV or Protein A, achieves the mitochondrial activity meanly 70% higher or comparable with the control on the tenth day of culture, which start from meanly 40% and 30%, respectively on the third day. A similar tendency represents PSS-Au alone, and with adsorbed COV or Protein A.

In the case of PEI, the membranes with adsorbed Protein A revealed fluctuations compared with the control: for PEI-Au + PROT A and PEI-Fe + PROT A, from the mitochondrial activity of about 30% control value on the third day to over twice on the tenth culture day; for PEI-Ag + PROT A, from mitochondrial activity comparable with control value on the third day to about 30% of control value on the tenth day of culture.

It cannot be excluded that ROS affects the MTT results. Response to stress caused by NP’s involvement may involve mitochondrial channels, whose activation may cause intra- and inter-mitochondrial redox environment changes, leading to ROS release [27]. The fluctuations of values observed in the MTT test may reflect this.

Some authors evaluated the influence on the cells of metallic nanoparticles, like AuNPs, AgNPs, copper oxide, and iron oxide nanoparticles. Collectively, it was observed that Au-NPs elicited mitochondrial dysfunction, leading to cell apoptosis and necrosis. However, AuNPs of different surface properties showed distinct effects on mitochondrial alterations, including reduced tubular mitochondria, damaged mitochondria, increased reactive oxygen species, and decreased adenosine triphosphate [28,29].

Reports about copper oxide and iron oxide nanoparticles showed similar effects as AuNPs, increasing cytotoxicity, mutagenicity, and mitochondrial impairment in the HepG2 cells [30].

Some authors evaluated in vivo results of the AgNP’s impact on a rodent model. It was reported that exposure to a low, environmentally relevant dose of AgNPs leads to induction of autophagy in adult rat brains in response to partial mitochondrial dysfunction. Moreover, the cells compensate for the defective autophagy mechanism by developing enhanced mitochondrial biodynamics [31].

The existing reports indicate that it is necessary to consider various parameters when designing modern nanoparticles.

The cytotoxicity of NPs towards biological material still needs further complex examinations in configuration with material predicted for antiviral application.

### 3.3. Analysis of the Adhesion of Cells Cultured in the Virus Presence

#### 3.3.1. Scanning Electron Microscopy Analysis

**Figure 6:**
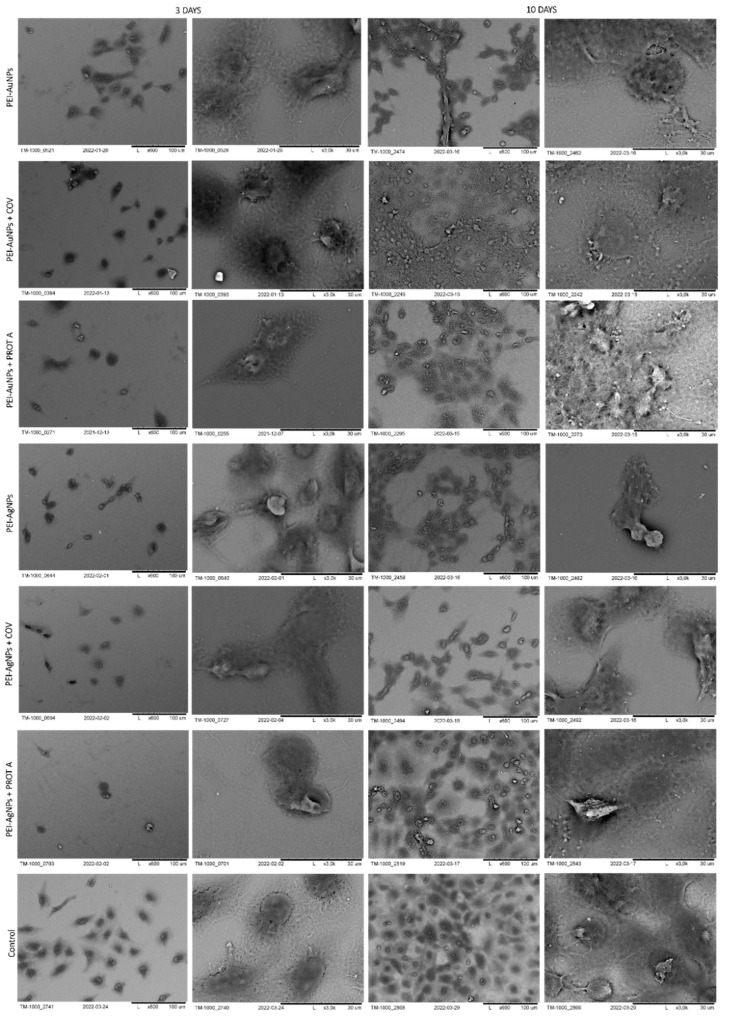
SEM visualization of A549 cells adsorbed within designed membranes after a 3- or 10-day culture. As a control, served cells cultured on glass slides without the membranes. Key to the symbols: PEI-AuNPs: the membrane build of the polyethylenimine with AuNPs incorporated; PEI-AuNPs + COV: the membrane build of the polyethylenimine with incorporated AuNPs with adsorbed COV; PEI-AuNPs + PROT A: the membrane build of the polyethylenimine with AuNPs incorporated with adsorbed Protein A; PEI-AgNPs: the membrane build of the polyethylenimine with AgNPs incorporated; PEI-AgNPs + COV: the membrane build of the polyethylenimine with AgNPs incorporated with adsorbed COV; PEI-AuNPs + PROT A: the membrane build of the polyethylenimine with AgNPs incorporated with adsorbed Protein A.

**Figure 7:**
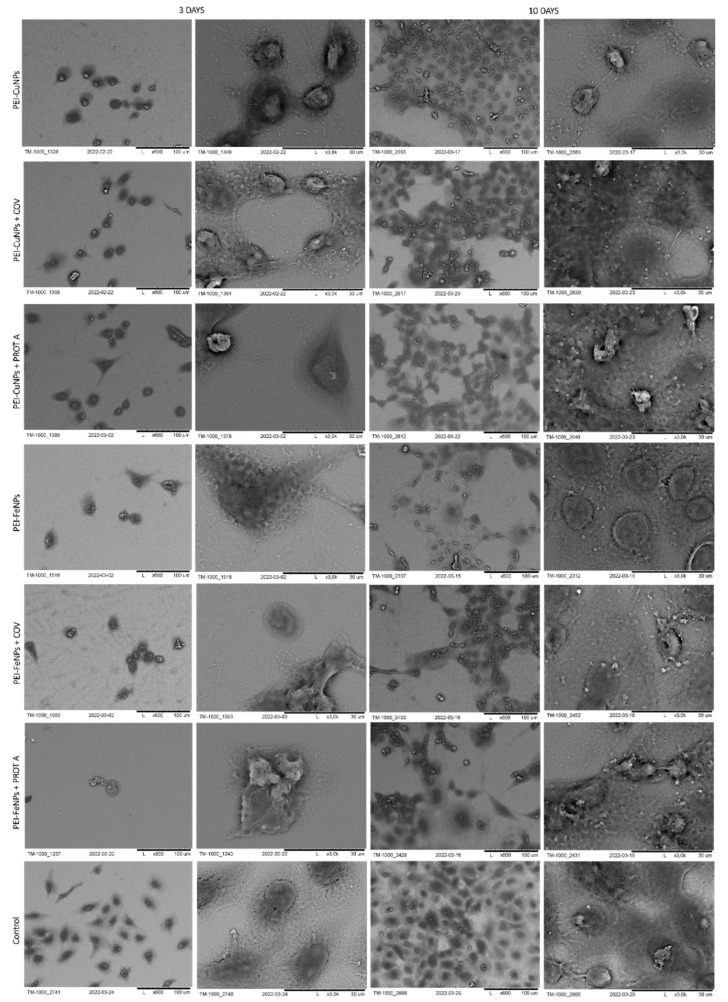
SEM visualization of A549 cells adsorbed within designed membranes after a 3- or 10-day culture. As a control, served cells cultured on glass slides without the membranes. Key to the symbols: PEI-AuNPs: the membrane build of the polyethylenimine with AuNPs incorporated; PEI-CuNPs: the membrane build of the polyethylenimine with CuNPs incorporated; PEI-CuNPs + COV: the membrane build of the polyethylenimine with CuNPs incorporated with adsorbed COV; PEI-CuNPs + PROT A: the membrane build of the polyethylenimine with CuNPs incorporated with adsorbed Protein A; PEI-FeNPs: the membrane build of the polyethylenimine with FeNPs incorporated; PEI-FeNPs + COV: the membrane build of the polyethylenimine with FeNPs incorporated with adsorbed COV; PEI-FeNPs + PROT A: the membrane build of the polyethylenimine with FeNPs incorporated with adsorbed Protein A.

**Figure 8:**
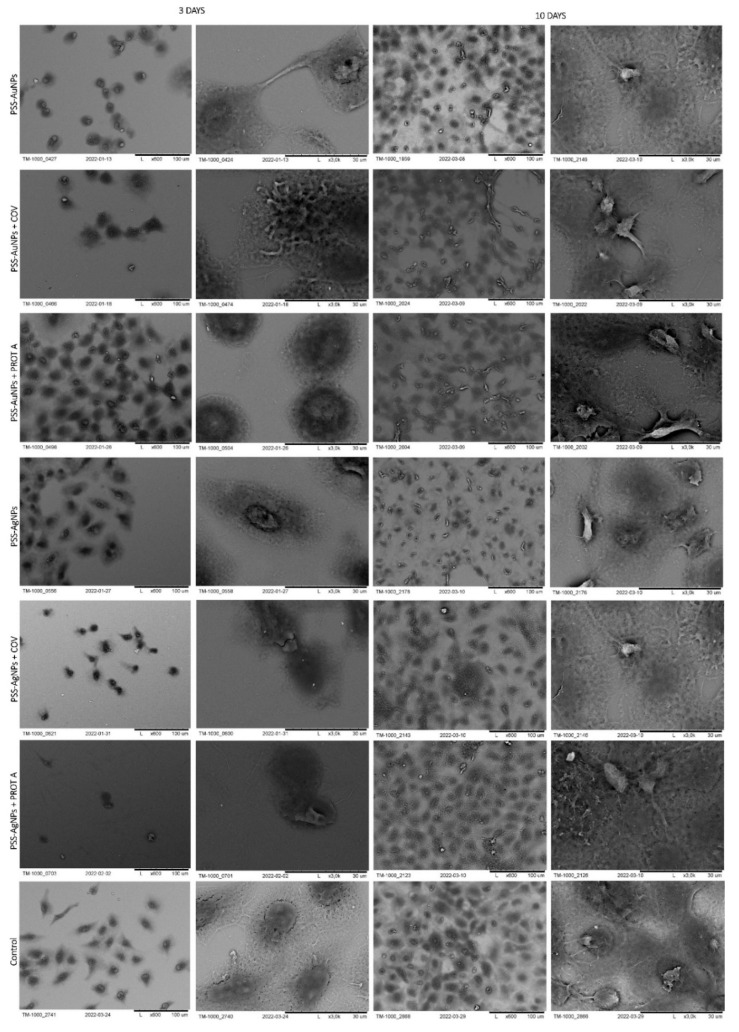
SEM visualization of A549 cells adsorbed within designed membranes after a 3- or 10-day culture. As a control, served cells cultured on glass slides without the membranes. Key to the symbols: PSS-AuNPs: the membrane build of the polystyrene sulfonate with AuNPs incorporated; PSS-AuNPs + COV: the membrane build of the polystyrene sulfonate with AuNPs incorporated with adsorbed COV; PSS-AuNPs + PROT A: the membrane build of the polystyrene sulfonate with AuNPs incorporated with adsorbed Protein A; PSS-AgNPs: the membrane build of the polystyrene sulfonate with AgNPs incorporated; PSS-AgNPs + COV: the membrane build of the polystyrene sulfonate with AgNPs incorporated with adsorbed COV; PSS-AuNPs + PROT A: the membrane build of the polystyrene sulfonate with AgNPs incorporated with adsorbed Protein A.

**Figure 9:**
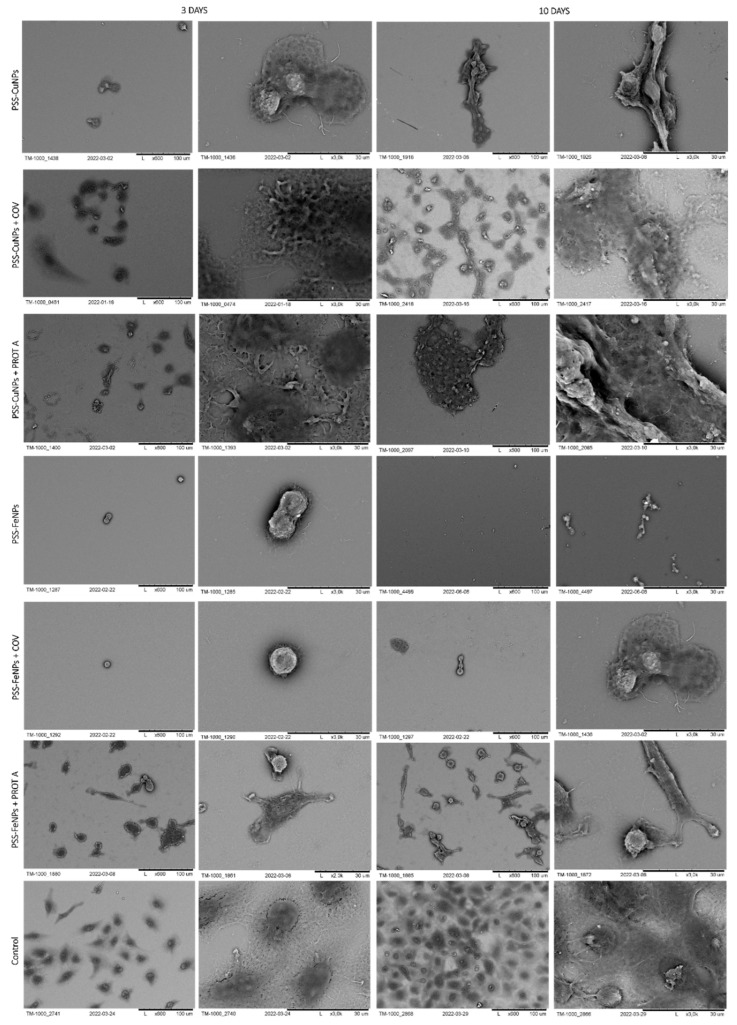
SEM visualization of A549 cells adsorbed within designed membranes after a 3- or 10-day culture. As a control, served cells cultured on glass slides without the membranes. Key to the symbols: PSS-CuNPs: the membrane build of the polystyrene sulfonate with CuNPs incorporated; PSS-CuNPs + COV: the membrane build of the polystyrene sulfonate with incorporated CuNPs with adsorbed COV; PSS-CuNPs + PROT A: the membrane build of the polystyrene sulfonate with CuNPs incorporated with adsorbed Protein A; PSS-FeNPs: the membrane build of the polystyrene sulfonate with FeNPs incorporated; PSS-FeNPs + COV: the membrane build of the polystyrene sulfonate with FeNPs incorporated with adsorbed COV; PSS-FeNPs + PROT A: the membrane build of the polystyrene sulfonate with FeNPs incorporated with adsorbed Protein A.

The morphology of cells immobilized within the developed membranes was examined using scanning electron microscopy (SEM), wherein the cells grown directly on a slide without membranes served as control. The images of the systems after 3 and 10 days of culture are shown in Figure 6, Figure 7, Figure 8 and Figure 9.

First, the membranes based on polyethyleneimine were analyzed, starting with the ones with incorporated gold nanoparticles. After 3 days of culture, the A549 cells adsorbed on PEI-AuNPs, PEI-AuNPs + COV, or PEI-AuNPs + PROT A membranes exhibited a spherical shape. Whereas, after 10 days of culture, the spherical shape A549 cells with some spindle ones were visible on the PEI-AuNPs and PEI-AuNPs + PROT A membranes. Furthermore, in the case of PEI-Au + PROT A, bunch-shaped structures were observed. Moreover, after 10 days of culture, an increase in the number of cells was observed for all membranes, with incorporated AuNPs. However, the cells on the surface of PEI-Au + PROT A membranes showed more intense growth than the PEI-AuNPs and PEI-AuNPs + COV.

Next, the observation was taken for the polyethyleneimine-based membranes with incorporated silver nanoparticles. In that case, after 3 days of culture, the A549 cells adsorbed on PEI-AgNPs or PEI-AgNPs + COV or PEI-AgNPs + PROT A membranes exhibited spherical shape cells with some number of spindle ones. After 10 days of culture, the observed morphology was similar. After 10 days of culture, growth in cell number was observed for all membranes with incorporated AgNPs. Nonetheless, the effect was the most visible for the cells on the surface of PEI-Ag + PROT A.

Similar to the polyethyleneimine-based membranes with golden and silver nanoparticles, the A549 cells adsorbed on PEI-CuNPs or PEI-CuNPs + COV or PEI-CuNPs + PROT A membranes exhibited a spherical shape after 3 days of maintenance. Additionally, after 10 days of culture, the A549 cells adsorbed on PEI-CuNPs or PEI-CuNPs + COV or PEI-CuNPs + PROT A detained the spherical shape. Furthermore, a rise in cell number was observed for all membranes with incorporated CuNPs after 10 days of culture, which showed similar growth patterns to the control group.

Finally, the polyethyleneimine membranes containing iron nanoparticles were studied. After 3 days of culture, the A549 cells adsorbed on PEI-FeNPs, PEI-FeNPs + COV, or PEI-FeNPs + PROT A membranes mostly showed a spherical shape; however a small number of spindle-shaped cells was also observed. As previously, after 10 days of culture, an increase in cell number was observed for all membranes with incorporated nanoparticles (here: FeNPs). In the case of PEI-Fe + PROT A, bunch-shaped structures could be observed. Compared to the control, the membranes on the base of PEI with incorporated FeNPs induce cell proliferation. On the other hand, the membranes on the base of PEI-Au or PEI-Fe with PROT A (PEI-Au + PROT A, PEI-Fe + PROT A) induce bunch-shaped structures of cells after 10 days of maintenance.

In the next stage, polystyrene sulfonate membranes were studied. First, the morphology of the ones with golden nanoparticles was tested. It was observed that, after 3 days of culture, the A549 cells adsorbed on PSS-AuNPs or PSS-AuNPs + COV or PSS-AuNPs + PROT A membranes showed a spherical shape. Moreover, the spherical shape cells, with some spindle-shaped ones, were visible for the A549 cells adsorbed on PSS-AuNPs or PSS-AuNPs + COV or PSS-AuNPs + PROT A membranes after 10 days of culture. The growth intensity was comparable for all the membranes incorporating AuNPs.

The observed cells’ morphology of the polystyrene sulfonate-based membranes with silver nanoparticles did not differ from the previous results. After 3 days of culture, the A549 cells adsorbed on PSS-AgNPs or PSS-AgNPs + COV or PSS-AgNPs + PROT A membranes exhibited a spherical shape; however, the single spindle cells were also present. After 10 days of culture, the observed morphology was alike. Moreover, an increase in cell number was noticed. The cells on the surface of PSS-Ag + PROT A membranes showed a number comparable with the control. The membranes PSS-Ag + PROT A induce bunch-shaped structures of cells after 10 days of culture.

Then, the polystyrene membranes with copper nanoparticles were verified. After 3 days of culture, the A549 cells adsorbed on PSS-CuNPs or PSS-CuNPs + COV or PSS-CuNPs + PROTA membranes exhibited a spherical shape. In addition, after 10 days of culture, the A549 cells adsorbed on PSS-CuNPs or PSS-CuNPs + COV or PSS-CuNPs + PROTA detained the spherical shape. The membranes PSS-Cu + PROT A induce bunch-shaped structures of cells after 10 days of culture.

Last but not least, polystyrene membranes incorporating iron nanoparticles were studied. After 3 and 10 days of culture, the A549 cells adsorbed on PSS-FeNPs or PSS-FeNPs + COV or PSS-FeNPs + PROT A membranes exhibited a spherical shape. Moreover, negligible cell proliferation was observed in the case of PSS-FeNPs or PSS-FeNPs + COV. Additionally, the cells adsorbed on PSS-FeNPs + PROT A showed a spherical shape, although the spindle cells were also visible.

It can be noted that the membranes on the base of PSS-Ag or PSS-Cu with PROT A induce bunch-shaped structures of cells after 10 days of culture.

#### 3.3.2. Atomic Force Microscopy Analysis

**Figure 10:**
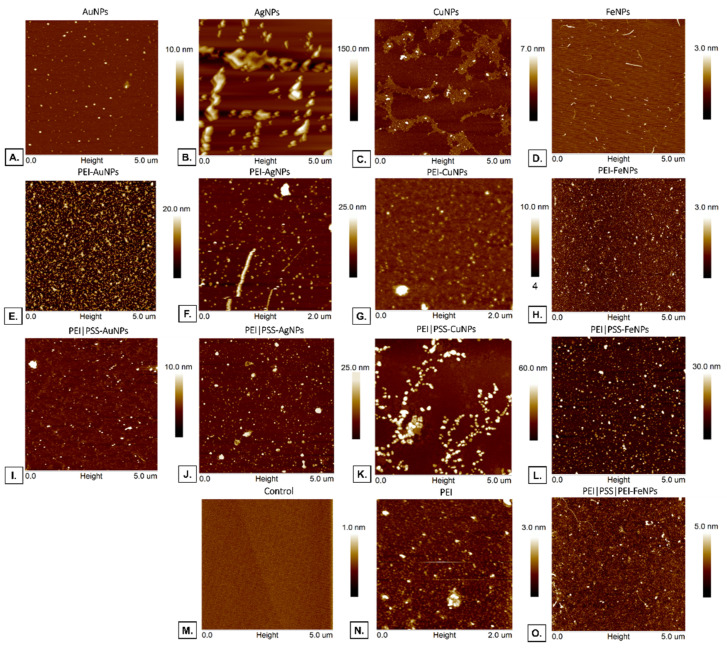
AFM image of polyelectrolyte membranes with incorporated nanoparticles deposited on the gold mica substrate cover. Key to the symbols: PEI-AuNPs: the membrane build of the polyethylenimine with AuNPs incorporated, PEI-AgNPs: the membrane build of the polyethylenimine with AgNPs incorporated, PEI-CuNPs: the membrane build of the polyethylenimine with CuNPs incorporated, PEI-FeNPs: the membrane build of the polyethylenimine with FeNPs incorporated; PEI|PSS-AuNPs: the membrane layer build of the polystyrene sulfonate with AuNPs incorporated deposited on polyethylenimine, PEI|PSS-AgNPs: the membrane build of the polystyrene sulfonate with AgNPs incorporated deposited on polyethylenimine, PEI|PSS-CuNPs: the membrane build of the polystyrene sulfonate with CuNPs incorporated deposited on polyethylenimine, PEI|PSS-FeNPs: the membrane build of the polystyrene sulfonate with FeNPs incorporated deposited on polyethylenimine, PEI: the polyethylenimine membrane, Control: mica surface.

Atomic Forces Microscopy (AFM) was performed to evaluate the material samples’ surface morphology. The image of polyelectrolyte membranes with incorporated nanoparticles is presented in Figure 10 The membranes PEI-FeNPs, PEI|PSS-FeNPs, and PEI|PSS|PEI-FeNPs (with root mean square average of profile height deviations from the mean line (R_ms_) [nm] respectively: 0.4; 3.9; 0.7) show an even structure over the entire surface with weakly marked active centers. The membranes PEI-AuNPs, PEI-AgNPs, and PEI-CuNPs (with R_ms_ [nm] 3.9; 3.3; 0.9, respectively) show an even structure over the entire surface with strongly marked active centers.

The membranes PEI|PSS-AuNPs (R_ms_ = 1.9 nm) exhibit a slightly branched structure with active centers; PEI/PSS-AgNPs (R_ms_ = 4.2 nm) show active centers on the surface, and PEI|PSS-CuNPs (R_ms_ = 9.7 nm), exhibit a strongly branched structure with active centers. The reflection of these structures can be seen in the SEM image in the cells’ pattern of the cells after 10 days of culture on the PSS-AuNPs, PSS-AgNPs, and PSS-CuNPs surface.

#### 3.3.3. Fourier Transform Infrared (FT-IR) Spectroscopy

**Figure 11:**
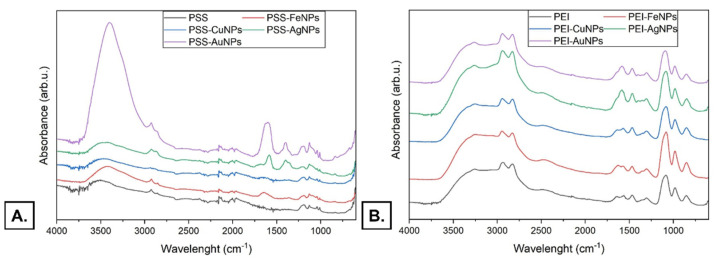
FTIR spectra of (**A**). polystyrene sulfonate and polystyrene sulfonate with AgNPs, AuNPs, CuNPs, FeNPs (**B**). polyethyleneimine and polyethyleneimine with AgNPs, AuNPs, CuNPs, FeNP. Key to the symbols: PSS—polystyrene sulfonate, PSS-AuNPs- polystyrene sulfonate with AuNPs, PSS-AgNPs- polystyrene sulfonate with AgNPs, PSS-CuNPs- polystyrene sulfonate with CuNPs; PSS-FeNPs- polystyrene sulfonate with FeNPs; PEI—polyethyleneimine, PEI-AuNPs polyethyleneimine with AuNPs, PEI-AgNPs polyethyleneimine with AgNPs, PEI-CuNPs polyethyleneimine with CuNPs, PEI-FeNPs polyethyleneimine with FeNPs.

The Fourier transform infrared spectra of the polystyrene sulfonate and polystyrene sulfonate with AgNPs, AuNPs, CuNPs, and FeNPs are presented in Figure 11A. The region showing characteristic peaks at 1012, 1037, 1125, and 1200 can be observed in the case of the PSS sample and PSS with AuNPs, AgNPs, CuNPs, and FeNPs. Stretching vibrations of the SO_3_ group appear as the 1037 and 1200 cm^−1^ peaks. Peaks at 1012 and 1125 cm^−1^ can be attributed to the vibration of the benzene ring [32]. The bands observed at 1625 cm^−1^ in PSS-FeNPs can be attributed to FeNPs’ presence. A very slight signal at the same wavelength might be observed for the PSS-CuNPs sample. The bands observed at 1600 cm^−1^ in the PSS-AuNPs sample can be attributed to the AuNPs’ presence. The PSS presence in the sample might cause dislocation of the band towards lower wavelength values compared with the value of about 1627 cm^−1^ for AuNPs alone [33]. The same was observed for PSS -AgNPs sample—the band appeared at 1575 cm^−1^. In contrast, the observed value for AgNPs alone is about 1630 cm^−1^ [34]. The lack of other visible bands distinguishing the samples containing PSS and NPs from the PSS itself may be due to the low concentration of the samples used in the experiments.

The Fourier transform infrared spectra of the polyethyleneimine and polyethyleneimine with AgNPs, AuNPs, CuNPs, and FeNP are presented in Figure 11B. In the given spectra, three regions can be distinguished. Three strong signals in the range of 800–1100 cm^−1^ can be assigned to the phosphate restudies from the buffer component [35]. The peaks in the 1250–1700 cm^−1^ can be assigned to various vibrations occurring in organic groups. Its structure is typical of PEI. The peak at 1450 cm^−1^ observed in the polyethyleneimine and polyethyleneimine with AgNPs, AuNPs, CuNPs, and FeNP samples can be attributed to N-H bond presence. Moreover, the N-H stretching modes can be observed in the region from 3000 to 3700 cm^−1^. In addition, the C-H bands of CH2 groups in PEI can be observed in the region from ~2800 to 2950 cm^−1^ [36]. The differences between the PEI sample and PEI with Fe NPs and CuNPs additions are minor. More changes can be observed for AuNPs and AgNPs samples. The band observed at 1588 cm^−1^ in the PEI-AuNPs sample can be attributed to the AuNPs’ presence. The PEI presence in the sample might cause dislocation of the band towards lower wavelength values compared with the value of about 1627 cm^−1^ for AuNPs alone [33]. Moreover, the band observed at the same wavelength of 1588 cm^−1^ in the PEI-AgNPs sample can be attributed to the AgNPs’ presence. The PEI presence in the sample might cause dislocation of the band towards lower wavelength values compared with the value for AgNPs alone, which is about 1630 cm^−1^ [34].

#### 3.3.4. Fluorescence Staining

**Figure 12:**
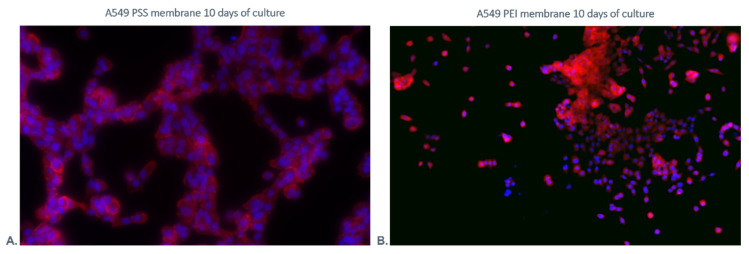
Visualization of cultured A549 cells grown on (**A**). PSS and (**B**). PEI membranes after 10 days of culture. The nuclei of cells are stained in blue. The red fluorescence shows F-Actin.

It was observed that the cells grown on PEI membranes presented clusters of dispersed cells. On the other hand, the cells cultured on the PSS membrane formed clusters in the form of chains (the representative pictures are shown in Figure 12). A correlation was noticed between the cell distribution pattern on the membrane surface and the surface pattern of the membrane layers assessed using AFM. It can be noted that the PSS layer induces a branched pattern on the surface.

## 4. Conclusions

As a result of COV adsorption to the PEI membrane surface, its morphology presents the shape of bunch-shaped structures. On the other hand, the COV adsorption to the PSS membrane surface implies flat morphology, with COV evenly dispersed all over the surface. Despite the different distribution patterns of COV on applied surfaces, both materials can act as COV-adsorbing layer nanocoating.

After 10-day culture, the membranes PEI-Au and PEI-Fe with adsorbed PROT A or COV and the membranes PSS-Au and PSS-Ag with adsorbed PROT A or COV do not exert cytotoxic activity, allowing the maintenance of lung cell function while maintaining morphology close to the control. Moreover, the membrane PSS-Fe + PROT A fulfills the above conditions. Likewise, the membrane PSS-Cu with adsorbed PROT A or COV fulfills the above conditions on the third day of culture.

AuNPs application seems to allow A549 to function in both configurations with the membrane of strongly negative and slightly positive potential [37], with the adsorbed virus or bacteria mimicking materials (PEI-Au + COV, PEI-Au + PROT A, PSS-Au + COV, PSS-Au + PROTA. However, no correlation was found between the membrane coating potential and its influence on cells.

The mean values of the percentage share of viable cells, cultured on membranes based on either PEI or PSS, incorporated with NPs alone or with COV and PROT A compared with the control differed by no more than 22% during 10 days of culture.

In general, PEI and PSS membrane layer coatings are modified with chosen metallic nanoparticles in configurations mentioned above that absorb COV or Protein A and support lung cells’ function, as evidenced by the results of the cytometric examination, confirmed by fluorescence microscopy and SEM visualization of the morphology of cells. However, no correlation was found between the cells’ viability and the cell distribution pattern on the membrane influenced by the surface pattern of membrane layers. Moreover, no correlation was found between the cells’ viability and roughness of either PEI or PSS membrane incorporated with NPs.

The designed material that absorbed COV or Protein A, which nevertheless does not adversely affect the cells of the lungs, can be recommended as material for biomedical applications, especially for personal protective and medical equipment surfaces, as it could reduce coronavirus spreading.

The obtained results may contribute to developing antiviral surfaces, although further research is necessary because no perfect anti-virus coating has been developed.

## Data Availability

Not applicable.
